# Oxygen injury in neonates: which is worse? hyperoxia, hypoxia, or alternating hyperoxia/hypoxia

**Published:** 2020-01-29

**Authors:** Tarek Mohamed, Amal Abdul-Hafez, Ira H Gewolb, Bruce D Uhal

**Affiliations:** 1 Department of Pediatrics and Human Development, Michigan State University, USA; 2 Department of Physiology, Michigan State University, USA

**Keywords:** Bronchopulmonary dysplasia, oxidative stress, renin angiotensin system, lung injury

## Abstract

Premature birth results in an increased risk of respiratory distress and often requires oxygen therapy. While the supplemental oxygen has been implicated as a cause of bronchopulmonary dysplasia (BPD), in clinical practice this supplementation usually only occurs after the patient’s oxygen saturation levels have dropped. The effect of hyperoxia on neonates has been extensively studied. However, there is an unanswered fundamental question: which has the most impact-hyperoxia, hypoxia or fluctuating oxygen levels? In this review, we will summarize the reported effect of hypoxia, hyperoxia or a fluctuation of oxygen levels (hypoxia/hyperoxia cycling) in preterm neonates, with special emphasis on the lungs.

## Bronchopulmonary dysplasia (BPD)

Bronchopulmonary dysplasia (BPD) is a form of chronic lung disease that affects premature newborns and infants and is the result of damage to the lungs caused by mechanical ventilation and long-term use of oxygen.^1–4^ Despite many advances in neonatal ventilation techniques and the widespread use of surfactant and antenatal corticosteroids, the incidence of BPD has been reported to be relatively stable at approximately 40% of surviving premature infants ≤28 weeks gestational age, with an estimated 10,000–15,000 new cases annually in the US alone.^5–11^ Mechanical ventilation and excessive oxygen supplementation are well-studied risk factors for BPD.^12–14^ Both the airway and parenchyma of the lung tissues are affected. These abnormalities have been attributed to ventilator induced injury as well as to oxygen therapy.^15^

BPD is recognized as a chronic lung disease of infancy that presents as a systemic syndrome and can be associated with neurodevelopmental deficits, cognitive impairments, failure to thrive, pulmonary hypertension and cor pulmonale.^16^ High rates of in utero and perinatal exposure to infection may be causally related to preterm delivery and subsequent lung injury.^17^ Over the past three decades, the histological presentation of BPD has changed from heterogeneous pulmonary inflammation and fibrosis (“Old BPD”) to uniform arrest of alveolar development and variable interstitial cellularity and/or fibroproliferation (“New BPD”).^18,19^

## Oxidative injury and development of BPD

### Reactive oxygen species (ROS) generation in the perinatal period

The fetus normally lives in a physiologically hypoxic environment, and relative hyperoxic exposure can cause an increase in the generation of “reactive oxygen species” (ROS). Preterm newborns are particularly vulnerable to oxygen toxicity due to inadequate levels of antioxidant enzymes, and hence to decreased protection from oxidative injury of rapidly growing tissues.^20–24^ The developing lung of the neonate is a perfect example of those vulnerable tissues; the endothelial cells and the alveolar type II cells are especially susceptible to oxidative injury. Oxidative stress in these cells activates transcription factors and pathways leading to cellular dysfunction, inactivation of surfactant, and impaired cell survival.^23–27^

Several pre- and post-natal adverse events generate ROS exposure and contribute to BPD pathogenesis; these include hypoxic, hyperoxic, and mechanical stimuli.^28^ Excess levels of oxygen (hyperoxia), as occur in supplemental oxygen administration, generate accumulation of ROS^1^. Hypoxia/hypoxemia, as in episodes of oxygen desaturations, also results in generation of ROS via a superoxide burst that occurs rapidly with hypoxic exposure.^29–31^ This ROS generation has been shown to be necessary for stabilization of Hypoxia Inducible Factor 1α (HIF-1α) and activation of the HIF system.^27,29,30^

**Figure 1:**
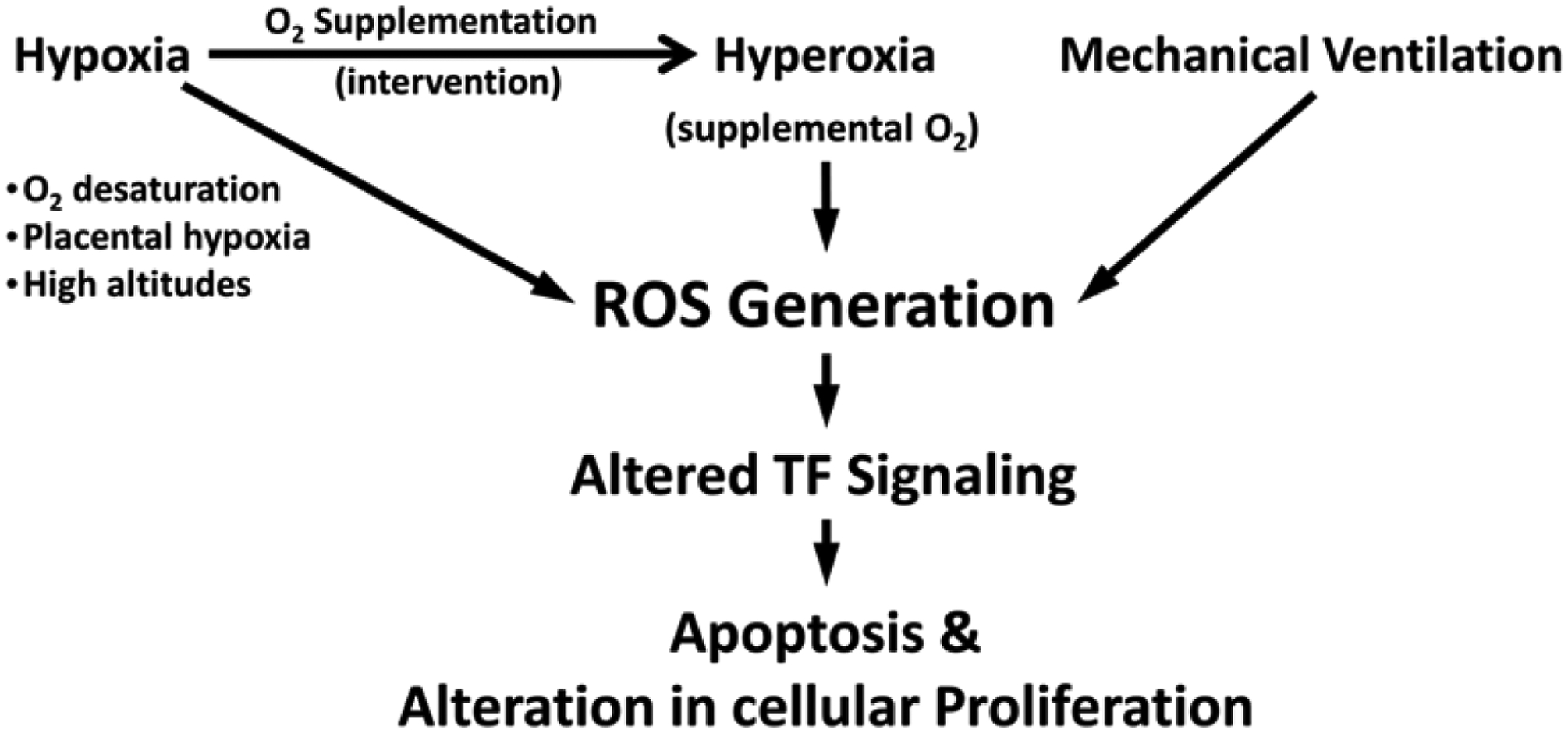
Schematic diagram illustrating reactive oxygen species (ROS) generation in neonatal pulmonary disease.

The ROS generated by both hyperoxia and hypoxia can result in alterations in cellular proliferation and apoptosis. Mechanisms may involve dysregulation of key transcription factors involved in ROS signaling such as HIF, nuclear factor E2 related factor 2 (Nrf2), NF-κB, and activator protein-1 (AP-1).^1,2,32^ Altered regulation of these transcription factors and pathways can disrupt postnatal alveolar development, cause inflammation, and potentially lead to fibrosis in preterm infants.^33,34^ ROS generation activates the HIF system.^27^ Nrf2 deficiency was shown to augment lung injury and arrest of alveolarization caused by hyperoxia during the newborn period.^32^ Both NF-κB and AP-1 are activated in multiple cell types, as well as in lung, following hyperoxia.^2^ A summary of ROS generation and neonatal lung injury is summarized in Figure 1.

### Prenatal hypoxia

Tissue injury due to oxidative stress is also noted in placental hypoxia, as is seen in preterm born neonates of pre-eclamptic mothers, where ROS are elevated and antioxidant levels are decreased in the maternal circulation.^26,35,36^ Placental hypoxia also induces an imbalance between pro-angiogenic and anti-angiogenic factors inhibiting VEGF signaling.^37,38^ This reduction in VEGF signaling in the developing lung was shown to result in impaired pulmonary vascular growth and alveolarization in neonatal rats.^39^ Maternal exposure to hypobaric hypoxia at high-altitudes can alter placental function, influence oxygen delivery to the fetus, result in lower birth weight, and increase the risk for pre-eclampsia.^40,41^

### Postnatal hypoxia

Premature babies are exposed post-natally to chronic or intermittent hypoxia as a result of immature lungs, apnea of prematurity, inadequate ventilation, or persistence of intrapulmonary arteriovenous shunts causing hypoxemia.^27,42^ Hypoxia of the infant leads to generalized pulmonary vasoconstriction and increases the pulmonary vascular resistance, causing pulmonary hypertension. Sustained hypoxic pulmonary vasoconstriction causes vascular remodeling of the pulmonary vascular bed leading ultimately to right heart failure.^43^ Postnatal hypoxia causes impaired alveolarization and alveolar simplification with fewer and larger alveoli. The impaired alveolarization also impairs the vascular maturation in the alveolar wall via mechanisms involving. altered signaling of HIF-1α, VEGF, and TGF-β,^27,44,45^

### Postnatal hyperoxic interventions

Preterm birth leads to premature transition of the pulmonary circulation from the physiologic hypoxic fetal environment to a relative hyperoxic postnatal environment (room air). In addition, oxygen supplementation is often required to fulfill the oxygen demands for adequate functioning of the body’s tissues and organs, in particular the brain, intestines, and kidneys.^28^ systemically circulating oxygen saturation levels (SPO_2_) is generally targeted to reach above 85%. However, the optimal systemic oxygen saturation in preterm infants is currently not clear. To compensate for the premature lung’s simplified alveolar structure and thick alveolar septae, higher levels of supplemental oxygen are often required to achieve the targeted intravascular oxygen levels, further augmenting the already existing (relative) postnatal hyperoxic state.^25,27^ Hyperoxia leads to impaired VEGF expression and disrupted angiogenesis and alveolarization due to rapid proteasomal degradation of HIF-1α.^46–48^ This induces vascular arrest, leading to pulmonary vascular diseases. Relative hyperoxia also increases generation of ROS and induces oxidative stress, an important contributor to the development of neonatal BPD. In addition, lower levels of antioxidants including vitamin E, transferrin, and superoxide dismutase, and higher levels of free iron further predispose preterm infants to oxidative injury.^20,21,23,49,50^

### Mechanical ventilation in BPD

Although mechanical ventilation is often essential and life-saving, it can provoke ventilator-induced lung injury in severely premature infants mainly by over-stretching of the distal epithelium and capillary endothelium.^25,51–55^ The development of that injury is dependent on the developmental stage of the lung, and the type, duration, volume and pressure of the mechanical ventilation.^52,54^ Mechanical ventilation also results in down regulation of VEGF-1 and its receptor flt-1 and up-regulation of the TGF-β co-receptor endoglin. This imbalance in mechanically ventilated lungs likely contributes to altered alveolarization and angiogenesis.^28,55,56^

## The renin angiotensin system (RAS) and BPD

### RAS and local tissue injury

**Figure 2:**
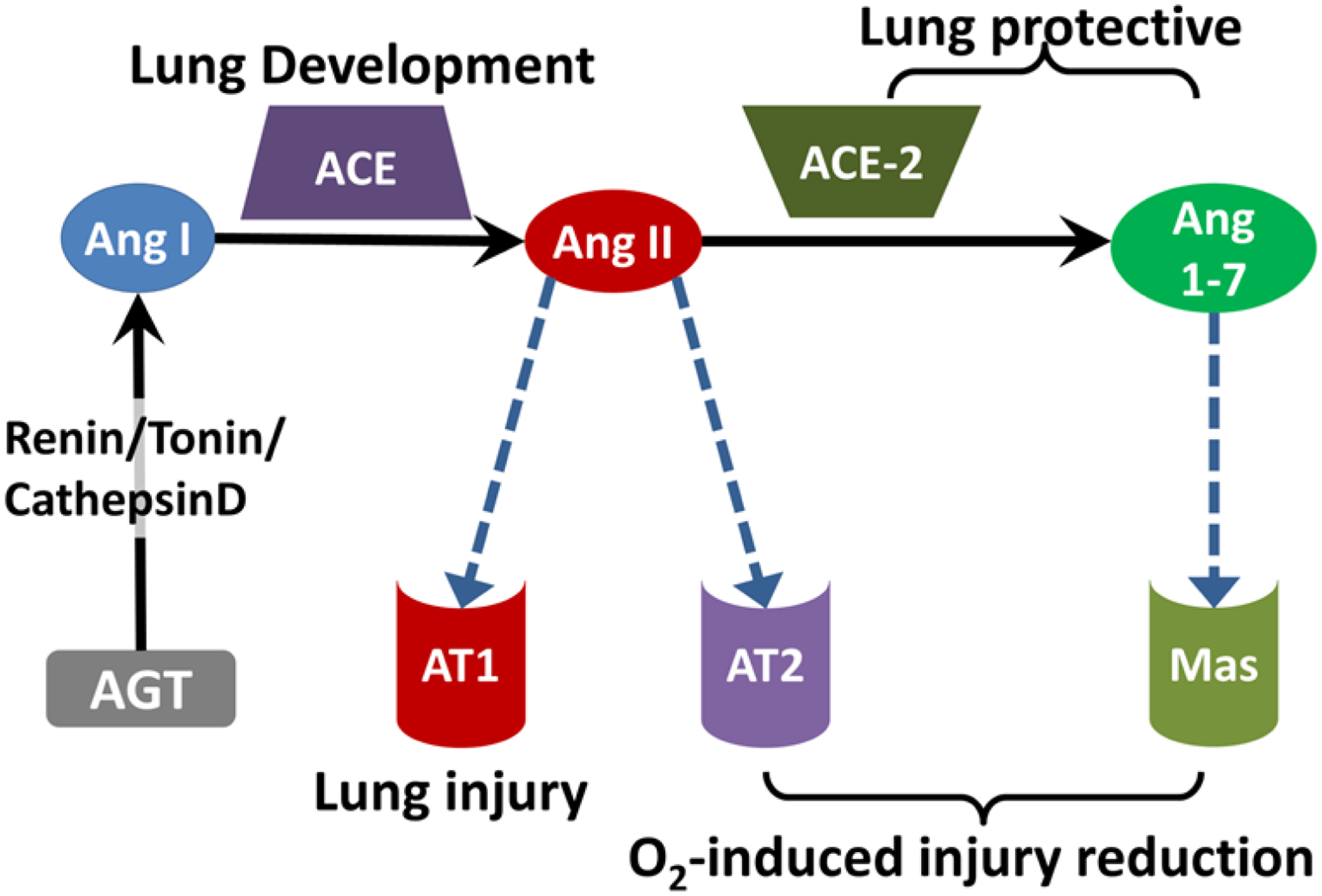
The renin angiotensin system components and its involvement in lung injury.

The RAS (Figure 2) is traditionally known to play a significant role in blood pressure regulation. Renin is produced by kidney and acts on circulating angiotensinogen (AGT) protein. Renin cleaves AGT to produce angiotensin I (Ang I). Ang I is converted by angiotensin-converting enzyme (ACE) to Ang II, which exerts its actions through binding to specific cell surface angiotensin receptors. Two main receptors to Ang II have been identified; AT_1_ and AT_2;_ both belong to a super family of seven transmembrane G-protein coupled receptors. The AT_1_ receptor mediates all of the classical actions of Ang II (vasodilatation, sodium retention, cell growth and proliferation), while the AT_2_ receptor promotes vasodilatation, cell differentiation, inhibition of cell growth and apoptosis and may play a counterbalancing role to the effects of Ang II on the AT_1_ receptor.^57^ ACE-2 and its product angiotensin 1–7 (Ang 1–7) acting on its receptor Mas were shown to have counteracting effects against the adverse actions of the other RAS components. Findings from numerous experimental studies have suggested notable protective effects of ANG1–7/Mas activation in the cardiovascular system.^58^ Local tissue effects of RAS have been identified in a variety of tissues such as heart, kidney, liver, lung, brain, pancreas and adipose tissue, where RAS component expression has been detected.^59,60^ Local RAS is involved in injury and inflammatory and fibrogenic diseases of many organs including heart,^61,62^ lung,^63–65^ liver,^66^ pancreas,^67^ and kidneys,^68,69^ by mechanisms independent of the blood-derived RAS.

### The role of RAS in BPD

RAS is believed to play a role in neonatal lung development and BPD pathogenesis (Figure 2). Perinatal exposure of animal models to ACE inhibitors was shown to disrupt normal alveolar and secondary septal formation during lung development in neonatal pups.^70^ In addition, neonatal rat ACE inhibition lowered the surface tension of bronchoalveolar lavage fluid and caused widening of respiratory airspaces and thinning of alveolar septa.^71^ Autopsy of human BPD patients showed decreased ACE expression in lungs as compared to controls without lung disease.^72^ Wagenaar et al.^4^ reported that Mas receptor and angiotensin receptor 2 (AT_2_) agonists reduced inflammation of oxygen-induced lung injury in rats. In this study, mRNA levels of the RAS component genes were measured during normal lung development. The mRNA levels of AT1, AT2, and ACE-2 decreased gradually, whereas expression of angiotensinogen and ACE-1 increased gradually as the neonatal rat lung develops. Exposure to 100% oxygen for 10 days resulted in an increase in expression of AT2 and a decrease in expression of AT1, angiotensinogen, and ACE.^4^ These studies suggest a critical role for angiotensin-angiotensin receptor signaling involving pulmonary alveolarization in the normal physiology of the neonatal lung and in the pathophysiology of BPD,. Angiotensinogen and ACE-1 play a role in alveolar development and septation, while the reported lower expression levels of ACE-2 in the neonatal lung could be facilitating the lung injury in the neonatal period.^4,70–72^

### The protective potential of ACE-2 in BPD

ACE-2 has been shown to play a protective role in lung disease through effects mediated by the receptor Mas, the receptor for the ACE-2 peptide product ANG1–7.^3,4,65,73^ Previous studies from our lab and other groups suggest that ACE-2 is down-regulated in fibrotic conditions of the adult and neonatal human lung,^3,65,73^ via Mas receptor mechanisms.^74^ We have also demonstrated that ACE-2 regulates alveolar epithelial cell survival by balancing the proapoptotic Ang II and its antiapoptotic degradation product Ang 1–7, through Ang 1–7 action on its receptor Mas.^74^ Furthermore, we showed that ACE-2 is expressed in fetal human lung fibroblasts but is significantly decreased by hyperoxic lung injury in a cell culture model.^3^ Importantly, this effect was reversed by hypoxia preceding hyperoxia.^65^ Recombinant human ACE-2 has been tested in healthy individuals in clinical trials to determine medication pharmacokinetics and pharmacodynamics,^75^ and has been investigated as pipeline drug “GSK2586881” in a pilot clinical trial to treat adult acute lung injury.^76^ ACE-targeted therapies might be future beneficial treatments for BPD.^77^

## Fluctuation of oxygen levels and BPD

Premature infants are known to experience intermittent episodes of hypoxemia lasting from a few seconds to several minutes^78^. Postnatal exposure to intermittent hypoxia followed by interventional hyperoxia induces oxidative stress and free radicals, which leads to direct cellular injury, oxidation of DNA, induction of cytokines, and recruitment of neutrophils and macrophages to the lung, manifested as pulmonary inflammation.^20,21^ Furthermore, ROS are released by immune cells resulting in epithelial and endothelial cell injury.^26^ Clinically unsuspected oxygen desaturation occurs frequently in preterm infants with and without bronchopulmonary dysplasia, and profound hypoxemia is claimed responsible for sudden unexplained deaths in these infants.^79^ Furthermore, infants who develop BPD experience more frequent episodes of oxygen desaturations than infants who recover from respiratory distress syndrome without developing BPD.^19,80^

## BPD and Fluctuation of oxygen levels in animal models

Very few studies in literature directly compare the effects of hyperoxia, hypoxia, or their fluctuation on the neonatal lung development or injury. Table 1 summarizes the studies discussed below. Ratner et al.^19^ demonstrated that cycling hypoxia with hyperoxia episodes exacerbated lung injury in neonatal mice.^19^ In their study they tested the effects of hypoxic episodes on a normoxia background, continuous hyperoxia, and hypoxic episodes combined with a hyperoxia background on neonatal mice and compared the results with the normoxia group. Compared with the normoxia control, the hypoxic episodes on normoxia background had no significantchanges on radial alveolar count (RAC) as a marker of lung injury, on oxidized glutathione, or on protein carbonyls as markers of oxidative stress, while continuous hyperoxia significantly reduced RAC and increased oxidative stress markers compared with normoxia. Interestingly, the intermittent hypoxia on hyperoxia background group of mice showed significantly lower RAC and higher oxidative stress markers compared with continuous hyperoxia. Their study suggested that the combination of two oxidative stress mechanisms, hypoxia and hyperoxia, cause a more profound lung injury than either one alone. Furthermore, their study even suggests that hypoxic mice did not have any BPD-like changes.^19^

A study by Schmiedl et al.^82^ on animal models of BPD compared the different effects of prenatal hypoxia and postnatal hyperoxia in neonatal mouse lung. Compared with normoxia controls, the lung volume, total air space volume and total septal surface were significantly reduced in the postnatal hyperoxia groups compared with either prenatal normoxia or prenatal hypoxia. The volume weighted mean volume of the parenchymal airspaces and the wall thickness of septa was significantly higher, and the volume density and the volume weighted mean volume of lamellar bodies in alveolar epithelial cells type II (AEII) were significantly lower in the prenatal hypoxia-postnatal hyperoxia group compared with normoxia controls, while prenatal normoxia-postnatal hyperoxia did not cause these changes. The study suggested that the prenatal hypoxia and postnatal hyperoxia model was found to best reflect morphological changes in lung development comparable with alterations found in BPD.^81^

A recent study by Valencia et al.^82^ examined the effects of intravitreal bevacizumab, a treatment for retinopathy of prematurity (ROP), on the lungs of a newborn rat pup model. The study showed long term effects of exposure to intermittent hypoxia and hyperoxia during the first 2 weeks of life. At postnatal days P23 and P45, both pO_2_ and SaO_2_ were lower in intermittent hypoxia exposure, while hyperoxia alone increased pO_2_ compared to the room air group. Interestingly, no significant difference was found in SaO_2_ in the hyperoxia group compared to room air.^82^ The results of this study suggest long-term effects of hypoxemia-hyperoxemia fluctuation on blood oxygenation and indicate the presence of a discrepancy between pO_2_ and SaO_2_ measures. In a 3-dimiensional cell culture organoid model of BPD, Sucre et al.^83^ showed that markers of fibroblast activation were increased by hypoxia-hyperoxia cycling as seen in BPD; α-SMA, Col1A1, TGFβ1, and PDE5a, and downstream targets of Wnt signaling (Cyclin D1, MMP2, and MMP9 RNA) were increased in hypoxia-hyperoxia relative to normoxia cultured organoids.^83^ A study by Chang et al.^84^ investigated the effects of hyperoxia with intermittent hypoxia on neonatal rats and found that repeated intermittent hypoxia during hyperoxia can alter biomarkers responsible for normal microvascular and alveolar development.^84^ However, both the Sucre et al.^83^ and Chang et al.^84^ studies have not compared the effects of hyperoxia alone to the effects of intermittent hypoxia during hyperoxia.

## What is the safe oxygen target level for newborns?

Several clinical studies and randomized controlled trials (RCTs) were performed to determine the range of optimal saturation by pulse oximetry in preterm infants receiving supplemental oxygen.^85–91^ Many of these studies have been discussed in detail in other reviews,^92,93^ we summarize these in Table 2. The assessment outcomes for these studies involved mortality, morbidity, development of chronic lung disease, and ROP. The current 2016 update of the European Consensus Guidelines on the management of neonatal respiratory distress syndrome recommends an oxygen saturation target between 90 and 94% with suggested alarm limits of 89 and 95%.^93^ These guides are based on the NeOProM meta-analysis study of the three largest and most recent RCTs^94^; the Surfactant Positive Pressure and Pulse Oximetry Randomized Trial (SUPPORT),^89^ the Benefits of Oxygen Saturation Targeting II (BOOST II),^90^ and the Canadian Oxygen Trial (COT).^91^ In these trials oxygen saturation monitoring by pulse oximetry was the method used to monitor oxygen levels. Pulse oximetry is currently the prevalent monitoring technology to detect blood oxygenation.^95^ However, invasive blood gas analysis is needed in order to monitor the oxygenation status when SpO2 is close to saturation (≥97%). A new technological measure using multiwavelength pulse co-oximetry called oxygen reserve index (ORI) can be used to monitor oxygenation in the moderate hyperoxemic range (PaO_2_ 100–200mmHg). The ORI is an index with a unit-less scale between 0.00 and 1.00, which is a relative indicator of changes in PaO2 in the moderate hyperoxemic range, and is used as a companion to pulse oximetry monitoring in patients receiving supplemental oxygen.^96^

## Effects of altered oxygen levels on other organs

### Retinopathy of prematurity

ROP is a disease affecting the development of the retinal vasculature characterized by abnormal growth of retinal blood vessels in the incompletely vascularized retina in preterm infants receiving supplemental oxygen therapy. ROP was first reported by Theodore L. Terry in 1942 and referred to as “retrolental fibroplasia”.^97,98^ An increase in arterial oxygen saturation, as when the preterm infant is resuscitated with high oxygen concentrations, is believed to be damaging to the newly developed retinal capillaries. After a preterm infant is no longer in supplemental oxygen, the avascular retina becomes hypoxic, leading to overexpression of angiogenic factors and vasoproliferation of intravitreal blood vessels.^99–101^ Penn et al.^102^ reported that exposure to variable hyperoxia (hypoxia/hyperoxia fluctuations) has been shown to be much more effective at producing proliferative retinopathy in neonatal rat than exposure to constant hyperoxia.^102^ Numerous animal studies established that intermittent hypox(em)ia cycling with hyperox(em)ia produces severe oxygen induced retinopathy.^82,103–107^ These studies emphasize the critical role of oxygen level fluctuations.

### Heart

Several independent studies in humans and animal models have reported that chronic fetal hypoxia can trigger a fetal origin of cardiac dysfunction and increase the risk of cardiovascular disease in later life.^108–115^ A transcriptomic study of neonatal ventricular chamber growth and development during perinatal circulatory transition identified Wnt11 as a prominent regulator of chamber-specific proliferation. Perinatal hypoxia treatment in mice suppressed Wnt11 expression and was associated with cyanotic congenital heart defect (CHD) phenotypes and correlated with O_2_ saturation levels in hypoxemic infants with Tetralogy of Fallot (TOF).^116^ Several animal studies have shown cardiovascular adverse effects of neonatal hyperoxia with long-term consequences into adulthood. Mouse model studies on perinatal or maternal inflammation combined with neonatal hyperoxia exposure showed altered fetal development affecting cardiac structure and function, resulting in early cardiac dysfunction leading to cardiac failure in adulthood.^117,118^ In newborn pigs, hyperoxia was found to trigger oxygen free radical-mediated membrane injury together with an inability of the newborn heart to up-regulate its antioxidant enzyme defenses while impairing myocardial function and hemodynamics.^119^ In addition, neonatal hyperoxia exposure was shown to increase systemic blood pressure and impair vasoreactivity in adult rats,^120^ possibly due to developmental programming of endothelial nitric oxide synthase uncoupling and enhanced vascular oxidative stress.^121^ Neonatal hyperoxia exposure also increased adult airway reactivity and was associated with left ventricular (LV) dysfunction in adult mice.^44^ Cardiac effects of hyperoxia also affect adults. In adult patients with and without congestive heart failure (CHF), hyperoxia was associated with impairment of cardiac relaxation and increased LV filling pressures. These studies indicate that caution should be used in the administration of high inspired O_2_ fractions.^122^

### Kidney

Oxygen supplementation and hyperoxic exposure of newborn animal models resulted in enlarged renal corpuscles and decreased number of nephrons in the kidneys in early adulthood.^120,123^ Neonatal hyperoxia exposure also resulted in impaired nephrogenesis causing reduction in both nephrogenic zone width and glomerular diameter and increased apoptotic cell count.^124^ Since the kidneys are highly vascular organs, the relative hyperoxia and oxygen supplementation in preterm infants could be responsible for the renal developmental abnormalities and for affecting glomerular vascularization, and should be examined in future studies.

## Summary of clinical importance/correlation

Various groups are shedding light on the importance of events that are commonly seen in neonatal ICUs, particularly with extremely low birth weight preterm babies, and with how prematurity affects the lung as well as other organs. Hyperoxia might not be the only risk factor for worsening BPD. However, uncontrollable fluctuation/cycling between hypoxemia, normoxemia and hyperoxemia might be a higher risk than hyperoxemia alone. There are trials of simulations to those cycling effects in different animal models. In the neonatal ICU there is no clear cut-off to delineate the “sweet-spot” in the oxygen saturation zone; how much is too much has not yet been clearly defined.

In mice, the episodic “fluctuation” of hypoxia and hyperoxia during the induction of BPD potentiated the oxidative stress in lung tissue and exacerbated the alveolar developmental arrest.^19^These results suggest that the aggressive prevention of hypoxemic episodes in human neonates at risk for the development of BPD needs to be further investigated. Our group, along with other groups, has studied the role of RAS in local tissue injury,^59,92,125–130^ and showed that ACE-2 might be beneficial to attenuate lung injury.^3,65,73^ A future study by our group is planned to look at the effect of cycling of hypoxemia/normoxemia/hyperoxemia and the effect of ACE-2 in a small animal model of BPD. Measurement of partial pressure of oxygen and/or SPO2 is planned to better assess and quantify the effects of hypoxemia with that of hyperoxemia/hypoxemia.

## Figures and Tables

**Table 1:** Summary of animal model studies for hypoxia-HYPEROXIA fluctuation (Cycling)

Reference	Organism	Cycling conditions		Cycling effect
		hypoxia	Hyperoxia	
Ratner et al.^19^	Mouse, start at P3	8% O2 (N2 balanced)	65% O2	• Fewer alveoli compared to HYPEROXIA
		10 min/episode*	All other times when not in episode	• Decreased pulmonary total/oxidized glutathione ratio (anti- oxidative capacity)
		1 episode/day for 1 Wk.+		• Elevation of protein carbonyl content (protein oxidation)
		1 episode/2days for 1 Wk.		
		*monitor SO_2_ by pulse oximetry		
		Total 4 Weeks		
Schmiedl et al.^81^	Mouse, start with pregnant mothers E14	10% O_2_	75% O_2_	• Higher volume of the parenchymal airspaces
		Pregnant mice	Pups	• Higher wall thickness of septa (not significant)
		E14->E18	P1->P14	• Lower volume of lamellar bodies in alveolar epithelial cells type II (by EM)
Valencia et al.^82^	Rat, start at P0	12% O_2_	50% O_2_	• At P23 and P45, compared with normoxia:
		1 min/episode	All other time	∘ Intermittent hypoxia decreased pO_2_
		3 episodes/cluster, 10 min apart		∘ HYPEROXIA increased pO2
		8 clusters/day for 2 Wks.		∘ No significant difference in SaO_2_ in the HYPEROXIA group
		Total 2 Weeks (P0->P14)		
Chang et al.^84^	Rat, start at P0 (within 5hr of birth)	12% O2	50% O2	• No HYPEROXIA only group was done
		2 min/episode	All other time	• No histology
		3 episodes/cluster, 10 min apart		• Measured VEGF, MMP2,9, TIMP by immunoassays and reversal of the effects by SOD mimetic
		4 clusters/day (every 6hr) for 2 Wks.		
		Total 2 Weeks (P0->P14)		
Sucre et al.^83^	3D organoid human fetal lung fibroblasts	10% O2	70% O2	• Markers of fibroblast activation were increased vs normoxia
		24 hr. then HYPEROXIA	24 hr. then hypoxia	• No comparison to hyperoxia alone made
		Total 4 days		

**Table 2:** Summary of clinical trials using oxygen saturation in neonatal care STOP-ROP, supplemental therapeutic oxygen for prethreshold retinopathy of prematurity; BOOST, benefits of oxygen saturation targeting; SUPPORT, surfactant positive pressure and pulse oximetry randomized trial; COT, canadian oxygen trial

Study (ref)	High SO_2_ level	Low SO_2_ Level	Outcome(s)
Tin and Gupta,^85^	88–98%	70–90%	Decreased incidence of ROP with low SO_2_ No differences in mortality and morbidity High SO_2_ had more cognitive disabilities after 10 years
Oxford Vermont Network,^86^	>95%	<95%	Less chronic lung disease and ROP incidence in low SO_2_ group
STOP-ROP,^87^	96–99%	89–94%	No significant difference in the rate of progression to threshold
BOOST I,^88^	95–98%	91–94%	Increased incidence of chronic lung disease and a longer duration of hospitalization with high SO_2_ No differences found in growth and neurodevelopmental measures at a corrected age of 12 months In the high SO_2_ group the newborns required oxygen for a longer period, had a higher dependence on oxygen at 36 weeks of postmenstrual age and needed home oxygen therapy with higher frequency
SUPPORT,^89^	91–95%	85–89%	No significant differences in severe ROP development, death before discharge from the hospital, or both Lower SO_2_ resulted in a decrease of occurrence of severe ROP Lower SO_2_ resulted in an increase of death before the discharge
BOOST II,^90^	91–95%	85–89%	Lower SO_2_ was associated with a higher risk of death and necrotizing enterocolitis Lower SO_2_ resulted a reduction of incidence of ROP
COT,^91^	91–95%	85–89%	No significant differences in death before 18 months of corrected age or survival with one or more disability Lower SO_2_ resulted in a reduction of duration of O2 therapy

## Abbreviations:

BPD: bronchopulmonary dysplasia
ROS: reactive oxygen species
HIF: hypoxia inducible factor
RAC: radial alveolar count
AP-1: activator protein-1
AGT: angiotensinogen
RCTs: randomized controlled trials
ACE: angiotensin-converting enzyme

## References

[1] Bhandari V Molecular mechanisms of hyperoxia-induced acute lung injury. Front Biosci. 2008;1:13:6653–6661. 10.2741/3179 18508685

[2] Lee PJ, Choi AM. Pathways of cell signaling in hyperoxia. Free Radic Biol Med. 2003;35(4):341–350. 12899937 10.1016/s0891-5849(03)00279-x

[3] Oarhe CI, Dang V, Dang M, Hyperoxia downregulates angiotensin-converting enzyme-2 in human fetal lung fibroblasts. Pediatr Res. 2015;77(5):656–62. 25665060 10.1038/pr.2015.27 PMC5119454

[4] Wagenaar GTM, Laghmani EH, Fidder M, Agonists of MAS oncogene and angiotensin II type 2 receptors attenuate cardiopulmonary disease in rats with neonatal hyperoxia-induced lung injury. Am J Physiol Lung Cell Mol Physiol. 2013;305(5):L341–51. 23812633 10.1152/ajplung.00360.2012 PMC3763032

[5] Smith VC, Zupancic JAF, McCormick MC, Trends in severe bronchopulmonary dysplasia rates between 1994 and 2002. J Pediatr. 2005;146(4):469–473. 15812448 10.1016/j.jpeds.2004.12.023

[6] Davidson LM, Berkelhamer SK. Bronchopulmonary Dysplasia: Chronic Lung Disease of Infancy and Long-Term Pulmonary Outcomes. J Clin Med. 2017;6(1). 10.3390/jcm6010004 PMC5294957 28067830

[7] Zysman-Colman Z, Tremblay GM, Bandeali S, Bronchopulmonary dysplasia - trends over three decades. Paediatr Child Health. 2013;18(2):86–90. 24421662 10.1093/pch/18.2.86 PMC3567902

[8] Lapcharoensap W, Gage SC, Kan P, Hospital Variation and Risk Factors for Bronchopulmonary Dysplasia in a Population-Based Cohort. JAMA Pediatr. 2015;169(2):e143676. 25642906 10.1001/jamapediatrics.2014.3676

[9] Stoll BJ, Hansen NI, Bell EF, Neonatal Outcomes of Extremely Preterm Infants From the NICHD Neonatal Research Network. Pediatrics. 2010;126(3):443–456. 20732945 10.1542/peds.2009-2959 PMC2982806

[10] Day CL, Ryan RM. Bronchopulmonary dysplasia: new becomes old again!. Pediatr Res. 2017;81(1–2):210–213. 27682969 10.1038/pr.2016.201

[11] Jensen EA, Schmidt B. Epidemiology of bronchopulmonary dysplasia. Birth Defects Res A Clin Mol Teratol. 2014;100(3):145–157. 24639412 10.1002/bdra.23235 PMC8604158

[12] Walsh MC, Yao Q, Gettner P, Impact of a physiologic definition on bronchopulmonary dysplasia rates. Pediatrics. 2004;114(5):1305–1311 15520112 10.1542/peds.2004-0204

[13] Deakins KM. Bronchopulmonary dysplasia. Respir Care. 2009;54(9):1252–1262. 19712501

[14] Berkelhamer SK, Mestan KK, Steinhorn RH. Pulmonary hypertension in bronchopulmonary dysplasia. Semin Perinatol. 2013;37(2):124–131. 23582967 10.1053/j.semperi.2013.01.009 PMC4464837

[15] Kair LR, Leonard DT, Anderson JM, et all. Bronchopulmonary Dysplasia. Pediatrics in Review. 2012;33(6):255–264. 22659256 10.1542/pir.33-6-255

[16] Northway WH, Rosan RC, Porter DY. Pulmonary disease following respirator therapy of hyaline-membrane disease. N Engl J Med. 1967;276(7):357–68. 5334613 10.1056/NEJM196702162760701

[17] Payne MS, Goss KCW, Connett GJ, Molecular microbiological characterization of preterm neonates at risk of bronchopulmonary dysplasia. Pediatr Res. 2010;67(4):412–8. 20035248 10.1203/PDR.0b013e3181d026c3

[18] Coalson JJ. Pathology of Bronchopulmonary Dysplasia. Semin Perinatol. 2006;30(4):179–184. 16860157 10.1053/j.semperi.2006.05.004

[19] Ratner V, Slinko S, Utkina-Sosunova I, Hypoxic stress exacerbates hyperoxia-induced lung injury in a neonatal mouse model of bronchopulmonary dysplasia. Neonatology. 2009;95(4):299–305. 19052476 10.1159/000178798 PMC3659784

[20] O’Donovan DJ, Fernandes CJ. Free Radicals and Diseases in Premature Infants. Antioxid Redox Signal. 2004;6(1):169–176. 14713348 10.1089/152308604771978471

[21] Ozsurekci Y, Aykac K. Oxidative Stress Related Diseases in Newborns. Oxid Med Cell Longev. 2016:1–9. 10.1155/2016/2768365 PMC4926016 27403229

[22] Saugstad OD. Mechanisms of tissue injury by oxygen radicals: implications for neonatal disease. Acta Paediatr. 1996;85(1):1–4. 8834970 10.1111/j.1651-2227.1996.tb13880.x

[23] Saugstad OD. Bronchopulmonary dysplasia-oxidative stress and antioxidants. Semin Neonatol. 2003;8(1):39–49. 12667829 10.1016/s1084-2756(02)00194-x

[24] Saugstad OD. Oxygen and oxidative stress in bronchopulmonary dysplasia. J Perinat Med. 2010;38(6):571–577. 20807008 10.1515/jpm.2010.108

[25] Stenmark KR, Abman SH. Lung vascular development: Implications for the Pathogenesis of Bronchopulmonary Dysplasia. Annu Rev Physiol. 2005;67(1):623–661. 15709973 10.1146/annurev.physiol.67.040403.102229

[26] Dennery PA. Role of Redox in Fetal Development and Neonatal Diseases. Antioxid Redox Signal. 2004;6(1):147–153. 14713346 10.1089/152308604771978453

[27] Vogel ER, Britt RD, Trinidad MC, Perinatal oxygen in the developing lung. Can J Physiol Pharmacol. 2015;93(2):119–127. 25594569 10.1139/cjpp-2014-0387 PMC4313769

[28] P D, Duncker DJ, Tibboel D, Oxidative injury of the pulmonary circulation in the perinatal period: Short- and long-term consequences for the human cardiopulmonary system. Pulm Circ. 2017;7(1):55–66. 28680565 10.1086/689748 PMC5448552

[29] Guzy RD, Schumacker PT. Oxygen sensing by mitochondria at complex III: the paradox of increased reactive oxygen species during hypoxia. Exp Physiol. 2006;91(5):807–819. 16857720 10.1113/expphysiol.2006.033506

[30] Hamanaka RB, Chandel NS. Mitochondrial reactive oxygen species regulate hypoxic signaling. Curr Opin Cell Biol. 2009;21(6):894–899. 19781926 10.1016/j.ceb.2009.08.005 PMC2787901

[31] Hernansanz-Agustín P, Izquierdo-Álvarez A, Sánchez-Gómez FJ, Acute hypoxia produces a superoxide burst in cells. Free Radic Biol Med. 2014;71:146–156. 24637263 10.1016/j.freeradbiomed.2014.03.011

[32] Cho H-Y, van Houten B, Wang X, Targeted Deletion of Nrf2 Impairs Lung Development and Oxidant Injury in Neonatal Mice. Antioxid Redox Signal. 2012;17(8):1066–1082. 22400915 10.1089/ars.2011.4288 PMC3423869

[33] Hilgendorff A, Reiss I, Ehrhardt H, Chronic Lung Disease in the Preterm Infant: Lessons Learned From Animal Models. Am J Respir Cell Mol Biol. 2013;50(2):233–245 10.1165/rcmb.2013-0014TR PMC5455410 24024524

[34] Madurga A, Mizikova I, Ruiz-Camp J, Recent advances in late lung development and the pathogenesis of bronchopulmonary dysplasia. Am J Physiol Lung Cell Mol Physiol. 2013;305(12):L893–L905. 24213917 10.1152/ajplung.00267.2013

[35] Namdev S, Bhat V, Adhisivam B, Oxidative stress and antioxidant status among neonates born to mothers with pre-eclampsia and their early outcome. J Matern Fetal Neonatal Med. 2014;27(14):1481–1484. 24188179 10.3109/14767058.2013.860521

[36] Negi R, Pande D, Karki K, Association of oxidative DNA damage, protein oxidation and antioxidant function with oxidative stress induced cellular injury in pre-eclamptic/eclamptic mothers during fetal circulation. Chem Biol Interact. 2014;208:77–83. 24296128 10.1016/j.cbi.2013.11.010

[37] Shah DA, Khalil RA. Bioactive factors in uteroplacental and systemic circulation link placental ischemia to generalized vascular dysfunction in hypertensive pregnancy and preeclampsia. Biochem Pharmacol. 2015;95(4):211–226. 25916268 10.1016/j.bcp.2015.04.012 PMC4449835

[38] Foidart JM, Schaaps JP, Chantraine F, Dysregulation of anti-angiogenic agents (sFlt-1, PLGF, and sEndoglin) in preeclampsia—a step forward but not the definitive answer. J Reprod Immunol. 2009;82(2):106–111. 19853925 10.1016/j.jri.2009.09.001

[39] Tang J-R, Karumanchi SA, Seedorf G, Excess soluble vascular endothelial growth factor receptor-1 in amniotic fluid impairs lung growth in rats: linking preeclampsia with bronchopulmonary dysplasia. Am J Physiol Lung Cell Mol Physiol. 2012;302(1):L36–L46. 22003089 10.1152/ajplung.00294.2011 PMC3349373

[40] Wilkins MR, Ghofrani H-A, Weissmann N, Pathophysiology and Treatment of High-Altitude Pulmonary Vascular Disease. Circulation. 2015;131(6):582–590. 25666980 10.1161/CIRCULATIONAHA.114.006977

[41] Mortola JP, Frappell PB, Aguero L, Birth weight and altitude: A study in Peruvian communities. J Pediatr. 2000;136(3):324–329. 10700688 10.1067/mpd.2000.103507

[42] Lovering AT, Riemer RK, Thébaud B. Intrapulmonary Arteriovenous Anastomoses. Physiological, Pathophysiological, or Both?. Ann Am Thorac Soc. 2013;10(5):504–508. 24161053 10.1513/AnnalsATS.201308-265ED

[43] Sommer N, Dietrich A, Schermuly RT, Regulation of hypoxic pulmonary vasoconstriction: basic mechanisms. Eur Respir J. 2008;32(6):1639–1651. 19043010 10.1183/09031936.00013908

[44] Ramani M, Bradley WE, Dell’Italia LJ, Early Exposure to Hyperoxia or Hypoxia Adversely Impacts Cardiopulmonary Development. Am J Respir Cell Mol Biol. 2015;52(5):594–602. 25255042 10.1165/rcmb.2013-0491OC PMC4491135

[45] Berger J, Bhandari V. Animal models of bronchopulmonary dysplasia. The term mouse models. Am J Physiol Lung Cell Mol Physiol. 2014;307(12):L936–L947. 25305249 10.1152/ajplung.00159.2014 PMC4269689

[46] Groenman F, Rutter M, Caniggia I, Hypoxia-inducible Factors in the First Trimester Human Lung. J Histochem Cytochem. 2007;55(4):355–363. 17189520 10.1369/jhc.6A7129.2006

[47] Rajatapiti P, de Rooij JD, Beurskens LWJE, Effect of Oxygen on the Expression of Hypoxia-Inducible Factors in Human Fetal Lung Explants. Neonatology. 2010;97(4):346–354. 20551700 10.1159/000261018

[48] Maxwell PH. Hypoxia-inducible factor as a physiological regulator. Exp Physiol. 2005;90(6):791–797. 16157658 10.1113/expphysiol.2005.030924

[49] Saugstad OD. Oxidative Stress in the Newborn – A 30-Year Perspective. Biol Neonate. 2005;88(3):228–236. 16210845 10.1159/000087586

[50] Rogers S, Witz G, Anwar M, Antioxidant capacity and oxygen radical diseases in the preterm newborn. Arch Pediatr Adolesc Med. 2000;154(6):544–548. 10850499 10.1001/archpedi.154.6.544

[51] Plötz FB, Slutsky AS, van Vught AJ, Ventilator-induced lung injury and multiple system organ failure: a critical review of facts and hypotheses. Intensive Care Med. 2004;30(10):1865–1872. 15221129 10.1007/s00134-004-2363-9

[52] Fan E, Villar J, Slutsky AS. Novel approaches to minimize ventilator-induced lung injury. BMC Med. 2013;11. 10.1186/1741-7015-11-85 PMC3621434 23536968

[53] Vlahakis NE, Schroeder MA, Limper AH, Stretch induces cytokine release by alveolar epithelial cells in vitro. Am J Physiol. 1999;277(1 Pt 1):L167–73. 10409244 10.1152/ajplung.1999.277.1.L167

[54] Jobe AH, Hillman N, Polglase G, Injury and Inflammation from Resuscitation of the Preterm Infant. Neonatology. 200;94(3):190–196. 18832854 10.1159/000143721

[55] De Paepe ME, Greco D, Mao Q. Angiogenesis-related gene expression profiling in ventilated preterm human lungs. Exp Lung Res. 2010;36(7):399–410. 20718599 10.3109/01902141003714031

[56] De Paepe ME, Patel C, Tsai A, Endoglin (CD105) Up-regulation in Pulmonary Microvasculature of Ventilated Preterm Infants. Am J Respir Crit Care Med. 2008;178(2):180–187. 18420967 10.1164/rccm.200608-1240OC PMC2453512

[57] Filippatos G, Tilak M, Pinillos H, Regulation of apoptosis by angiotensin II in the heart and lungs (Review). Int J Mol Med. 2001;7(3):273–80. 11179507 10.3892/ijmm.7.3.273

[58] Jiang F, Yang J, Zhang Y, Angiotensin-converting enzyme 2 and angiotensin 1–7: novel therapeutic targets. Nat Rev Cardiol. 2014;11(7):413–26. 24776703 10.1038/nrcardio.2014.59 PMC7097196

[59] Li X, Zhang H, Soledad-Conrad V, Bleomycin-induced apoptosis of alveolar epithelial cells requires angiotensin synthesis de novo. Am J Physiol Lung Cell Mol Physiol. 2003;284(3):L501–L5017. 12573988 10.1152/ajplung.00273.2002

[60] Filippatos G, Uhal BD. Blockade of apoptosis by ACE inhibitors and angiotensin receptor antagonists. Curr Pharm Des. 2003;9(9):707–714. 12570788 10.2174/1381612033455477

[61] Hale TM. Persistent phenotypic shift in cardiac fibroblasts: impact of transient renin angiotensin system inhibition. J Mol Cell Cardiol. 2016;93:125–32. 26631495 10.1016/j.yjmcc.2015.11.027

[62] Simões E Silva AC, Teixeira MM. ACE inhibition, ACE2 and angiotensin-(1–7) axis in kidney and cardiac inflammation and fibrosis. Pharmacol Res. 2016;107:154–62. 26995300 10.1016/j.phrs.2016.03.018

[63] Uhal BD, Li X, Piasecki CC, Angiotensin signalling in pulmonary fibrosis. Int J Biochem Cell Biol. 2012;44(3):465–468. 22155301 10.1016/j.biocel.2011.11.019 PMC3288339

[64] Uhal BD, Abdul-Hafez A. Angiotensin II in apoptotic lung injury: potential role in meconium aspiration syndrome. J Perinatol. 2008;28 Suppl 3:S108–12. 19057599 10.1038/jp.2008.149

[65] Mohamed TL, Nguyen HT, Abdul-Hafez A, Prior hypoxia prevents downregulation of ACE-2 by hyperoxia in fetal human lung fibroblasts. Exp Lung Res. 2016;42(3):121–130. 27093376 10.3109/01902148.2016.1157712 PMC4915782

[66] Grace JA, Herath CB, Mak KY, Update on new aspects of the renin-angiotensin system in liver disease: clinical implications and new therapeutic options. Clin Sci (Lond). 2012;123(4):225–239. 22548407 10.1042/CS20120030

[67] Goossens GH. The renin-angiotensin system in the pathophysiology of type 2 diabetes. Obes Facts. 2012;5(4):611–624. 22986649 10.1159/000342776

[68] Macconi D, Remuzzi G, Benigni A. Key fibrogenic mediators: old players. Renin-angiotensin system. Kidney Int Suppl (2011). 2014;4(1):58–64. 26312151 10.1038/kisup.2014.11 PMC4536968

[69] Chappell MC, Abadir P, Foster D, Biochemical evaluation of the renin-angiotensin system: the good, bad, and absolute?. Am J Physiol Heart Circ Physiol. 2016;310(2):H137–52. 26475588 10.1152/ajpheart.00618.2015 PMC4796631

[70] Capelari DN, Sánchez SI, Ortega HH, Effects of maternal captopril treatment during late pregnancy on neonatal lung development in rats. Regul Pept. 2012;177(1–3):97–106. 22587910 10.1016/j.regpep.2012.05.092

[71] Lasaitiene D, Chen Y, Nannmark U, Neonatal ACE inhibition in rats interferes with lung development. Clin Physiol Funct Imaging. 2004;24(1):65–68. 14717750 10.1046/j.1475-0961.2003.00530.x

[72] Castro EC, Parks WT, Galambos C. The ontogeny of human pulmonary angiotensin-converting enzyme and its aberrant expression may contribute to the pathobiology of bronchopulmonary dysplasia (BPD). Pediatr Pulmonol. 2014;49(10):985–990. 24574430 10.1002/ppul.22911

[73] Li X, Molina-Molina M, Abdul-Hafez A, Angiotensin converting enzyme-2 is protective but downregulated in human and experimental lung fibrosis. Am J Physiol Lung Cell Mol Physiol. 2008;295(1):L178–85. 18441099 10.1152/ajplung.00009.2008 PMC2494775

[74] Uhal BD, Li X, Xue A, Regulation of alveolar epithelial cell survival by the ACE-2 / angiotensin 1 – 7 / Mas axis. Am J Physiol Lung Cell Mol Physiol. 2011;779(3):269–274. 10.1152/ajplung.00222.2010 PMC3174737 21665960

[75] Haschke M, Schuster M, Poglitsch M, Pharmacokinetics and pharmacodynamics of recombinant human angiotensin-converting enzyme 2 in healthy human subjects. Clin Pharmacokinet. 2013;52(9):783–792. 23681967 10.1007/s40262-013-0072-7

[76] Khan A, Benthin C, Zeno B, A pilot clinical trial of recombinant human angiotensin-converting enzyme 2 in acute respiratory distress syndrome. Crit Care. 2017;21(1):234. 28877748 10.1186/s13054-017-1823-x PMC5588692

[77] Gandhi C, Uhal BD. Roles of the Angiotensin System in Neonatal Lung Injury and Disease. JSM Atheroscler. 2016;1(3):1014. 29806050 PMC5967852

[78] Martin RJ, Wang K, Köroğlu O, Intermittent hypoxic episodes in preterm infants: do they matter?. Neonatology. 2011;100(3):303–310. 21986336 10.1159/000329922 PMC3252018

[79] Garg M, Kurzner SI, Bautista DB, Clinically Unsuspected Hypoxia During Sleep and Feeding in Infants With Bronchopulmonary Dysplasia. Pediatrics. 1988;81(5):635–642. 3357725

[80] Jia L, Xu M, Zhen W, Novel anti-oxidative role of calreticulin in protecting A549 human type II alveolar epithelial cells against hypoxic injury. Am J Physiol Cell Physiol. 2008;294(1):C47–C55. 17959730 10.1152/ajpcell.00019.2007

[81] Schmiedl A, Roolfs T, Tutdibi E, Influence of prenatal hypoxia and postnatal hyperoxia on morphologic lung maturation in mice. PLoS ONE. 2017;12(4):1–21. 10.1371/journal.pone.0175804 PMC5398543 28426693

[82] Valencia AM, Cai CL, Tan J, Intravitreal bevacizumab alters type IV collagenases and exacerbates arrested alveologenesis in the neonatal rat lungs. Exp Lung Res. 2017;43(3):1–14. 28409646 10.1080/01902148.2017.1306897

[83] Sucre JMS, Vijayaraj P, Aros CJ, Posttranslational modification of β-catenin is associated with pathogenic fibroblastic changes in bronchopulmonary dysplasia. Am J Physiol Lung Cell Mol Physiol. 2017;312(2):L186–L195. 27941077 10.1152/ajplung.00477.2016 PMC5336582

[84] Chang M, Bany-Mohammed F, Cristina Kenney M, Effects of a superoxide dismutase mimetic on biomarkers of lung angiogenesis and alveolarization during hyperoxia with intermittent hypoxia. Am J Transl Res. 2013;5(6):594–607. 24093057 PMC3786267

[85] Tin W, Gupta S. Optimum oxygen therapy in preterm babies. Arch Dis Child Fetal Neonatal Ed. 2007;92(2):F143–F147. 17337663 10.1136/adc.2005.092726 PMC2675464

[86] Sun S Relation of target SpO (2) levels and clinical outcome in ELBW infants on supplemental oxygen. Pediatric Research. 2002.

[87] Supplemental Therapeutic Oxygen for Prethreshold Retinopathy of Prematurity (STOP-ROP), A Randomized, Controlled Trial. I: Primary Outcomes. Pediatrics. 2000;105(2):295–310. 10654946 10.1542/peds.105.2.295

[88] Askie LM, Henderson-Smart DJ, Irwig L, Oxygen-Saturation Targets and Outcomes in Extremely Preterm Infants. N Engl J Med. 2003;349(10):959–967. 12954744 10.1056/NEJMoa023080

[89] Carlo WA, Finer NN, Walsh MC, Target Ranges of Oxygen Saturation in Extremely Preterm Infants. N Engl J Med. 2010;362(21):1959–1969. 20472937 10.1056/NEJMoa0911781 PMC2891970

[90] Stenson BJ, Tarnow-Mordi WO, Darlow BA, Oxygen Saturation and Outcomes in Preterm Infants. N Engl J Med. 2013;368(22):2094–2104. 23642047 10.1056/NEJMoa1302298

[91] Schmidt B, Whyte RK, Asztalos EV, Effects of Targeting Higher vs Lower Arterial Oxygen Saturations on Death or Disability in Extremely Preterm Infants : a randomized clinical trial. JAMA. 2013;309(20):2111–2120 23644995 10.1001/jama.2013.5555

[92] Perrone S, Bracciali C, Di Virgilio N, Oxygen Use in Neonatal Care: A Two-edged Sword. Front Pediatr. 2017;4:143. 28119904 10.3389/fped.2016.00143 PMC5220090

[93] Sweet DG, Carnielli V, Greisen G, European Consensus Guidelines on the Management of Respiratory Distress Syndrome - 2016 Update. Neonatology. 2017;111(2):107–125. 27649091 10.1159/000448985

[94] Askie LM, Brocklehurst P, Darlow BA, NeOProM: Neonatal Oxygenation Prospective Meta-analysis Collaboration study protocol. BMC Pediatr. 2011;11(1):6. 21235822 10.1186/1471-2431-11-6 PMC3025869

[95] Jubran A Pulse oximetry. Crit Care. 2015;19(1):272. 26179876 10.1186/s13054-015-0984-8 PMC4504215

[96] Scheeren TWL, Belda FJ, Perel A. The oxygen reserve index (ORI): a new tool to monitor oxygen therapy. J Clin Monit Comput. 2018;32(3):379–389. 28791567 10.1007/s10877-017-0049-4 PMC5943373

[97] Terry TL. Fibroblastic Overgrowth of Persistent Tunica Vasculosa Lentis in Infants Born Prematurely: II. Report of Cases-Clinical Aspects. Trans Am Ophthalmol Soc.1942;40:262–284. 16693285 PMC1315050

[98] Coats DK, Reddy AK. Retinopathy of Prematurity. In: Wilson ME, Trivedi RH, Saunders RA, editors. Pediatric Ophthalmology: Current Thought and A Practical Guide. Berlin, Heidelberg: Springer Berlin Heidelberg; 2009. p. 375–386.

[99] Hartnett ME, Lane RH. Effects of oxygen on the development and severity of retinopathy of prematurity. J AAPOS. 2013;17(3):229–234. 23791404 10.1016/j.jaapos.2012.12.155 PMC3740273

[100] Flynn JT, Bancalari E, Snyder ES, A cohort study of transcutaneous oxygen tension and the incidence and severity of retinopathy of prematurity. Trans Am Ophthalmol Soc. 1991;89:77–92. 1808822 PMC1298617

[101] Hartnett ME. Studies on the pathogenesis of avascular retina and neovascularization into the vitreous in peripheral severe retinopathy of prematurity (an american ophthalmological society thesis). Trans Am Ophthalmol Soc. 2010;108:96–119. 21212851 PMC3016082

[102] Penn JS, Henry MM, Tolman BL. Exposure to Alternating Hypoxia and Hyperoxia Causes Severe Proliferative Retinopathy in the Newborn Rat. Pediatr Res. 1994;36(6):724–731. 7898981 10.1203/00006450-199412000-00007

[103] Coleman RJ, Beharry KDA, Brock RS, Effects of Brief, Clustered Versus Dispersed Hypoxic Episodes on Systemic and Ocular Growth Factors in a Rat Model of Oxygen-Induced Retinopathy. Pediatr Res. 2008;64(1):50–55. 18344903 10.1203/PDR.0b013e31817307ac

[104] Brock RS, Gebrekristos BH, Kuniyoshi KM, Biomolecular Effects of Jb1 (an IGF-I Peptide Analog) in a Rat Model of Oxygen-Induced Retinopathy. Pediatric Research. 2011;69(2):135–141. 21057375 10.1203/PDR.0b013e318204e6fa

[105] Beharry KD, Cai CL, Sharma P, Hydrogen peroxide accumulation in the choroid during intermittent hypoxia increases risk of severe oxygen-induced retinopathy in neonatal rats. Invest Ophthalmol Vis Sci. 2013;54(12):7644–7657. 24168990 10.1167/iovs.13-13040 PMC3835271

[106] Jivabhai Patel S, Bany-Mohammed F, McNally L, Exogenous Superoxide Dismutase Mimetic Without Scavenging H2O2 Causes Photoreceptor Damage in a Rat Model for Oxygen-Induced Retinopathy. Invest Ophthalmol Vis Sci. 2015;56(3):1665–1677. 25670494 10.1167/iovs.14-15321 PMC4354243

[107] Aranda JV, Cai CL, Ahmad T, Pharmacologic synergism of ocular ketorolac and systemic caffeine citrate in rat oxygen-induced retinopathy. Pediatr Res. 2016;80(4):554–565. 27438224 10.1038/pr.2016.105 PMC5030702

[108] Itani N, Skeffington KL, Beck C, Niu Y, Giussani DA. Melatonin rescues cardiovascular dysfunction during hypoxic development in the chick embryo. J Pineal Res. 2016;60(1):16–26. 26444711 10.1111/jpi.12283 PMC4832387

[109] Veille J, Hanson R, Sivakoff M, Fetal Cardiac Size in Normal, Intrauterine Growth Retarded, and Diabetic Pregnancies. Am J Perinatol. 1993;10(04):275–279. 8397561 10.1055/s-2007-994739

[110] Zhang L Prenatal Hypoxia and Cardiac Programming. J Soc Gynecol Investig. 2005;12(1):2–13. 10.1016/j.jsgi.2004.09.004 15629664

[111] Williams SJ, Hemmings DG, Mitchell JM, Effects of maternal hypoxia or nutrient restriction during pregnancy on endothelial function in adult male rat offspring. J Physiol. 2005;565(Pt 1):125–35. 15774515 10.1113/jphysiol.2005.084889 PMC1464495

[112] Akira M, Yoshiyuki S. Placental Circulation, Fetal Growth, and Stiffness of the Abdominal Aorta in Newborn Infants. J Pediatr. 2006;148(1):49–53. 16423597 10.1016/j.jpeds.2005.06.044

[113] Camm EJ, Hansell JA, Kane AD, Partial contributions of developmental hypoxia and undernutrition to prenatal alterations in somatic growth and cardiovascular structure and function. Am J Obstet Gynecol. 2010;203(5):495.e24–495.e34. 10.1016/j.ajog.2010.06.046 20708165

[114] Giussani DA, Camm EJ, Niu Y, Developmental Programming of Cardiovascular Dysfunction by Prenatal Hypoxia and Oxidative Stress Calbet JAL, editor. PLoS ONE. 2012;7(2):e31017. 22348036 10.1371/journal.pone.0031017 PMC3278440

[115] Thompson LP, Al-Hasan Y. Impact of Oxidative Stress in Fetal Programming. J Pregnancy. 2012;2012:1–8. 10.1155/2012/582748 PMC3403156 22848830

[116] Touma M, Kang X, Gao F, Wnt11 regulates cardiac chamber development and disease during perinatal maturation. JCI Insight. 2017;2(17). 10.1172/jci.insight.94904 PMC5621892 28878122

[117] Velten M, Gorr MW, Youtz DJ, Adverse perinatal environment contributes to altered cardiac development and function. American journal of physiology. Am J Physiol Heart Circ Physiol. 2014;306(9):H1334–40. 24610916 10.1152/ajpheart.00056.2014 PMC4010669

[118] Velten M, Hutchinson KR, Gorr MW, Systemic maternal inflammation and neonatal hyperoxia induces remodeling and left ventricular dysfunction in mice. PloS one. 2011;6(9):e24544. 21935422 10.1371/journal.pone.0024544 PMC3173376

[119] Bandali KS, Belanger MP, Wittnich C. Hyperoxia causes oxygen free radical-mediated membrane injury and alters myocardial function and hemodynamics in the newborn. Am J Physiol Heart Circ Physiol. 2004;287(2):H553–H559. 15277198 10.1152/ajpheart.00657.2003

[120] Yzydorczyk C, Comte B, Cambonie G, Neonatal Oxygen Exposure in Rats Leads to Cardiovascular and Renal Alterations in Adulthood. Hypertension. 2008;52(5):889–895. 18852387 10.1161/HYPERTENSIONAHA.108.116251

[121] Yzydorczyk C, Comte B, Huyard F, Developmental Programming of eNOS Uncoupling and Enhanced Vascular Oxidative Stress in Adult Rats After Transient Neonatal Oxygen Exposure. J Cardiovasc Pharmacol. 2013;61(1):8–16. 23011469 10.1097/FJC.0b013e318274d1c4

[122] Mak S, Azevedo ER, Liu PP, Effect of hyperoxia on left ventricular function and filling pressures in patients with and without congestive heart failure. Chest. 2001;120(2):467–73. 11502645 10.1378/chest.120.2.467

[123] Sutherland MR, O’Reilly M, Kenna K, Neonatal hyperoxia: effects on nephrogenesis and long-term glomerular structure. Am J Physiol Renal Physiol. 2013;304(10):F130–16. 10.1152/ajprenal.00172.2012 23427140

[124] Popescu CR, Sutherland MR, Cloutier A, Hyperoxia Exposure Impairs Nephrogenesis in the Neonatal Rat: Role of HIF-1α Ashton N, editor. PLoS ONE. 2013;8(12):e82421. 24358181 10.1371/journal.pone.0082421 PMC3866112

[125] Uhal BD, Joshi I, True AL, Fibroblasts isolated after fibrotic lung injury induce apoptosis of alveolar epithelial cells in vitro. Am J Physiol. 1995;269(6 Pt 1):L819–28. 8572243 10.1152/ajplung.1995.269.6.L819

[126] Wang R, Zagariya A, Ibarra-Sunga O, Angiotensin II induces apoptosis in human and rat alveolar epithelial cells. Am J Physiol. 1999;276(5 Pt 1):L885–9. 10330045 10.1152/ajplung.1999.276.5.L885

[127] Papp M, Li X, Zhuang J, Angiotensin receptor subtype AT(1) mediates alveolar epithelial cell apoptosis in response to ANG II. Am J Physiol Lung Cell Mol Physiol. 2002;282(4):L713–8. 11880296 10.1152/ajplung.00103.2001

[128] Li X, Molina-Molina M, Abdul-Hafez A, Extravascular sources of lung angiotensin peptide synthesis in idiopathic pulmonary fibrosis. Am J Physiol Lung Cell Mol Physiol. 2006;291(5):L887–95. 16844946 10.1152/ajplung.00432.2005

[129] Hillman NH, Gisslen T, Polglase GR, Ventilation-induced increases in EGFR ligand mRNA are not altered by intra-amniotic LPS or ureaplasma in preterm lambs. PloS one. 2014;9(4):e96087. 24788984 10.1371/journal.pone.0096087 PMC4005755

[130] Poisner AM, Adler F, Uhal B, Persistent and progressive pulmonary fibrotic changes in a model of fat embolism. J Trauma Acute Care Surg. 2012;72(4):992–998. 22491616 10.1097/TA.0b013e31823c96b0

